# Evidence for Tissue Toxicity in BALB/c Exposed to a Long-Term Treatment with Oxiranes Compared to Meglumine Antimoniate

**DOI:** 10.1155/2017/9840210

**Published:** 2017-07-17

**Authors:** Luiz Filipe Gonçalves Oliveira, Franklin Souza-Silva, Léa Cysne-Finkelstein, Kíssila Rabelo, Juliana Fernandes Amorim, Adriana de Souza Azevedo, Saulo Cabral Bourguignon, Vitor Francisco Ferreira, Marciano Viana Paes, Carlos Roberto Alves

**Affiliations:** ^1^Laboratório de Biologia Molecular e Doenças Endêmicas, Instituto Oswaldo Cruz, Fundação Oswaldo Cruz, Avenida Brasil 4365, Manguinhos, 21040-900 Rio de Janeiro, RJ, Brazil; ^2^Laboratório de Imunoparasitologia, Instituto Oswaldo Cruz, Fundação Oswaldo Cruz (Fiocruz), Avenida Brasil 4365, Manguinhos, 21040-900 Rio de Janeiro, RJ, Brazil; ^3^Laboratório de Ultraestrutura e Biologia Tecidual, Instituto de Biologia Roberto Alcantara Gomes, Universidade do Estado do Rio de Janeiro, Boulevard 28 de Setembro, No. 87, Vila Isabel, 20551-030 Rio de Janeiro, RJ, Brazil; ^4^Laboratório de Tecnologia Virológica, Instituto de Tecnologia em Imunobiológicos, Fundação Oswaldo Cruz (Fiocruz), Avenida Brasil 4365, Manguinhos, 21040-900 Rio de Janeiro, RJ, Brazil; ^5^Instituto de Biologia, Universidade Federal Fluminense, Outeiro São João Batista S/N, 24210-130 Niterói, RJ, Brazil; ^6^Departamento de Química Orgânica, Instituto de Química, Universidade Federal Fluminense, Outeiro São João Batista S/N, Centro, 24210-130 Niterói, RJ, Brazil; ^7^Laboratório Interdisciplinar de Pesquisas Médicas, Instituto Oswaldo Cruz, Fundação Oswaldo Cruz, Avenida Brasil 4365, Manguinhos, 21040-900 Rio de Janeiro, RJ, Brazil

## Abstract

Leishmaniasis remains a serious public health problem in developing countries without effective control, whether by vaccination or chemotherapy. Part of the failure of leishmaniasis control is due to the lack of new less toxic and more effective drugs able to eliminate both the lesions and the parasite. Oxiranes derived from naphthoquinones now being assayed are promising drugs for the treatment of this group of diseases. The predicted pharmacokinetic properties and toxicological profiles of epoxy-*α*-lapachone and epoxymethoxy-lawsone have now been compared to those of meglumine antimoniate, and histological changes induced by these drugs in noninfected BALB/c mice tissues are described. Effects of these compounds on liver, kidney, lung, heart, and cerebral tissues of healthy mice were examined. The data presented show that both these oxiranes and meglumine antimoniate induce changes in all BALB/c mice tissues, with the lung, heart, and brain being the most affected. Epoxymethoxy-lawsone was the most toxic to lung tissue, while most severe damage was caused in the heart by epoxy-*α*-lapachone. Meglumine antimoniate caused mild-to-moderate changes in heart and lung tissues.

## 1. Introduction

Leishmaniasis represents a group of parasitic diseases caused by more than 20* Leismania* spp. parasites, which are transmitted to humans by the bite of infected female phlebotomine sandflies. The epidemiology of leishmaniasis depends on the characteristics of the parasite species, the local ecological characteristics of the transmission sites, current and past exposure of the human population to the parasite, and human behavior [1]. Different forms of leishmaniasis represent an important public health problem in the Americas, due to their widespread distribution and high prevalence. Besides that, the risk factors of transmission are linked to socioeconomic and environmental patterns, which make it difficult to control the disease. Human infections result in a variety of clinical symptoms involving the skin, the mucosa of the upper respiratory tract, and the visceral organs, with difficult treatment for severe cases and with little assurance of parasitological cure [2].

Leishmaniasis treatment was initially described in 1912 by Vianna, when the curative action of a trivalent antimonial (Sb^3+^) on the skin form of the disease was discovered [3]. However, due to their severe toxic side effects trivalent antimonials were replaced after 1940 by pentavalent antimonials (Sb^5+^), which have remained even nowadays as the drug of choice for the treatment of all forms of leishmaniasis. In cases where Sb^5+^ treatment is unsuccessful, second-choice drugs, as amphotericin B and pentamidine, are used [4]. The wide variety of adverse effects attributed to these drugs is well described in the literature, ranging from clinical complaints such as musculoskeletal pain and nausea and vomiting to serious toxic effects on heart, liver, and kidney [5]. The most hazardous side effect associated with antimonials is undoubtedly cardiotoxicity, characterized by several changes in the cardiovascular system, particularly altered ventricular repolarization [reviewed in [6]]. Also increasing resistance to pentavalent antimonials has been reported requiring the use of higher doses and longer-term therapy, increasing the toxic risk [7, 8]. Drug resistance has been reported in India, with patients failing to respond or showing a relapse after therapy with antimony drugs [9]. Resistance to the current therapy with antimony drug has also been reported in many other countries including Afghanistan, Iran, Brazil, Peru, and Saudi Arabia [10].

In this context, the search for novel less toxic antileishmanial drugs has been the subject of several studies. Interest in applying herbal extracts, essential oils, and natural products against leishmaniasis has increased in recent years [11]. Many research groups have searched for new potential antiparasitic drugs from vegetable sources, mainly based on secondary metabolites, of which naphthoquinones are an example [12].

Quinones are a wide and varied family of natural aromatic metabolites found in several plant families, as well as in fungi, algae, and bacteria. These quinones include benzoquinones, anthraquinones, and naphthoquinones [13]. An important chemical property of quinones is their ability to act as oxidizing or dehydrogenating agents. This redox property is driven by the formation of a fully aromatic system [14, 15]. In recent years, interest in quinones and their derivatives has increased not only due to role in biochemical processes but also for their activity against a wide range of infectious agents [16]. In nature, these compounds, such as ubiquinones (coenzyme Q) and menaquinones (vitamin K), are involved in important stages of the life cycle, acting primarily as components of the electron transport chains and in photosynthesis [17].

**Figure 1:**
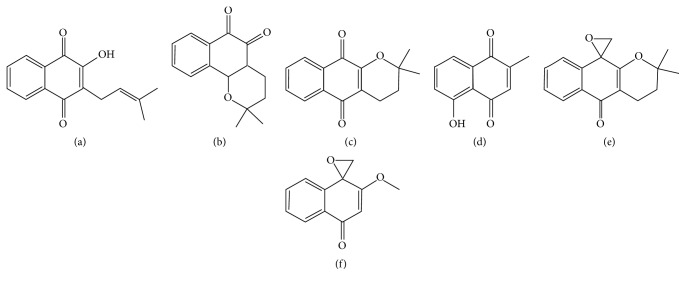
Chemical structure of main active naphthoquinones and oxiranes. (a) Lapachol (CID 3884; molecular formula, C_15_H_14_O_3_; molecular weight, 242.274 g/mol); (b) *β*-lapachone (CID 3885; molecular formula, C_15_H_14_O_3_; molecular weight, 242.274 g/mol); (c) *α*-lapachone (CID 72732; molecular formula, C_15_H_14_O_3_; molecular weight, 242.274 g/mol); (d) plumbagin (CID 10205; molecular formula, C_11_H_8_O_3_; molecular weight, 188.182 g/mol); (e) epoxy-*α*-lapachone (CID 12000280; molecular formula, C_16_H_16_O_3_; molecular weight, 256.301 g/mol); (f) epoxymethoxy-lawsone (molecular formula, C_12_H_10_O_3_; molecular weight, 202.210 g/mol) (https://pubchem.ncbi.nlm.nih.gov/compound/12000280#section=Top).

1,4-Naphthoquinones are derived from naphthalene by the introduction of two carbonyl groups in the 1,4 positions or, less commonly, in the 1,2 positions (1,2-naphthoquinones) [13]. Their interest in medicinal chemistry derives from their ability to control infection by and multiplication of trypanosomatids of medical interest [18]. Examples of quinones which have been described as effective against* Leishmania* species and* Trypanosoma cruzi* include lapachol (Figure 1(a)), *β*-lapachone (Figure 1(b)), and *α*-lapachone (Figure 1(c)), first isolated from the heartwood of trees of the Bignoniaceae family (*Tabebuia* sp.), and plumbagin (Figure 1(d)), extracted from* Plumbago scandens* L. roots and other* Plantago *spp. [19–21].

However, the toxicity of the lapachone derivatives limits their potential for use in the treatment in leishmaniasis and other diseases. Chemical modifications were performed on these compounds to address this issue and modification of the quinonoid center of *α*-lapachone followed by epoxidation generated the epoxy-*α*-lapachone (Figure 1(e)) and epoxymethoxy-lawsone (Figure 1(f)), derivatives potentially less toxic for mammalian cells [22, 23].

Epoxy-*α*-lapachone led to a dose- and time-dependent decrease in the numbers of promastigotes of* Leishmania (Viannia) braziliensis* and* Leishmania (Leishmania) amazonensis*. This compound was able to kill amastigotes inside human macrophages [24]. In addition, this reduction of the lesion size in the paw of BALB/c mice infected by* L. (L.) amazonensis* was observed until six weeks after treatment and negatively affected the activity of serine proteinase of both promastigote and amastigote forms [25].

The potential use of these oxiranes derived from natural 1,4-naphtoquinones against* Leishmania* parasites to treat the infection in humans made it relevant to evaluate the prediction of pharmacokinetic properties and possible toxic effects of epoxy-*α*-lapachone and epoxymethoxy-lawsone comparing to meglumine antimoniate and describe histological changes induced by these drugs in noninfected BALB/c mice tissues. Additionally, the possible biochemical mechanisms associated with cell damage in these tissues are discussed. The data gathered here aggregate new evidences on histological changes in mammalian host tissues induced by oxiranes and meglumine antimoniate.

## 2. Materials and Methods

### 2.1. Chemicals and Culture Reagents

Dimethyl sulfoxide (DMSO), penicillin, streptomycin, and Schneider's Drosophila medium were purchased from Sigma-Aldrich Chemical Co. (St. Louis, MO). Fetal calf serum (FCS) was acquired from Cultilab S/A (Brazil). Meglumine antimoniate (Glucantime) was purchased from Sanofi-Aventis Farmacêutica (Suzano, Brazil). Propylene glycol was obtained from Vetec Quimica. Epoxy-*α*-lapachone and epoxymethoxy-lawsone compounds were synthesized by the Department of Organic Chemistry of the Instituto de Química, Universidade Federal Fluminense.

### 2.2. In Silico Analysis

In silico assessing of the pharmacokinetics and toxicity parameters of oxiranes compounds and meglumine antimoniate was performed here. Pharmacokinetic parameters (absorption, distribution, metabolism, and excretion) and toxicological were tested using the web server platform “pkCSM: predicting small-molecule pharmacokinetic properties using graph-based signatures” (available in http://bleoberis.bioc.cam.ac.uk/pkcsm/prediction). Data on absorption, distribution, metabolism, excretion (ADME), and toxicity (T) of those compounds are presented in Table 1.

### 2.3. Parasite Cultures

*Leishmania (Leishmania) amazonensis* (MHOM/BR/73/LTB0016) was obtained from the Leishmania collection of the Instituto Oswaldo Cruz (Fiocruz). In vitro promastigote cultures were maintained at 28°C in Schneider's medium (pH 7.2) containing 1 mM L-glutamine, 10% FCS, 100 IU/mL penicillin, and 100 *μ*g/mL streptomycin, with frequent subpassages to maintain the parasites in the logarithmic growth phase. The parasites were in the stationary growth phase after 5 days of culture in Schneider's medium.

### 2.4. Experimental Treatments

All experiments were conducted with 5-to-7-week-old BALB/c mice weighing approximately 22 g. The animals were obtained from the animal breeding center of Fiocruz, and all experimental procedures were performed as approved by the Committee for the Ethical Use of Animals of Instituto Oswaldo Cruz (L-052/2015). A group of BALB/c mice were inoculated subcutaneously, in the left footpad, with 1.0 × 10^4^ promastigotes and the lesion sizes were measured weekly. Treatments of infected and noninfected mice were performed with either meglumine antimoniate (Glucantime) as a comparative control for treatment efficacy 22.7 mg/Kg/day (4.1 *μ*M of Sb^5+^) or epoxy-*α*-lapachone at 22.7 mg/Kg/day (1.9 *μ*M/Kg/day) and epoxymethoxy-lawsone at 11.4 mg/Kg/day (1.2 *μ*M/Kg/day) and diluted in a mix of DMSO : propylene glycol : saline (1 : 12: 7 and 1 : 9 : 10, resp.). The drugs were administered subcutaneously in the dorsal region of each mouse in a dose of 100 *μ*L per animal. Negative-control groups were included, in which sterile PBS or the dilution mix of DMSO : propylene glycol : saline with higher concentration of propylene glycol (1 : 12 : 7) was administered during treatment. All treatments were made daily from Monday to Friday, until 20 doses.

### 2.5. Histopathological Study

Organs (liver, lung, heart, kidney, and brain/cerebellum) were collected from six BALB/c mice of each group (drugs, vehicle, or no treatment). Fragments of the tissues were excised, cleaved, fixed in buffered formalin (10%), and blocked in paraffin. Tissue sections, 4 *μ*m thick, were cut, deparaffinized in three baths of xylene, and rehydrated with increasing concentrations of ethanol (70% to 100%). Hematoxylin and eosin (HE) were used finally to stain the tissue sections for histological examination. Tissue damage of liver, lung, heart, brain, and cerebellum were semiquantitatively assessed based on intensity and focal or diffuse character. A total of 10 fragments of tissue for each animal were examined and results were plotted as the media of damage values. For each lesion a parameter was assigned with a numerical value between 0 and 4, according to the severity and extent of the damage: 0 = absent, 1 = mild, 2 = moderate, 3 = severe, and 4 = severe-diffuse. The tissue alterations quantified in the liver were circulatory changes (hemorrhage and edema) and inflammatory infiltrates in the central and portal vein and necrosis. For quantification of lung tissue alterations, circulatory alterations, the thickening of the alveolar septa, and bronchiolar inflammatory infiltrates were considered. In the case of kidney tissue, measurements took into account circulatory changes (hemorrhage, edema, and vascular congestion); cortical infiltrates; disorganization of the cortical area and regions of the collecting, proximal, and distal tubules; and destructed epithelial lining of distal convoluted tubules. Tissue injuries were measured over the whole slide to be quantified by optical microscopy Olympus BX53 using Image Pro Plus software and all analyses were performed in a blind manner without prior knowledge of the group.

## 3. Results

### 3.1. In Silico Pharmacokinetic and Toxicity Parameters of Oxirane Compounds and Meglumine Antimoniate

A computational study was performed for the prediction of ADME profile of each of the oxiranes derived from 1,4-naphthoquinones as well as meglumine antimoniate and the main results are presented in Table 1. Both oxiranes are completely absorbed by the oral and intestinal mucosa, as demonstrated by Caco-2 monolayer test and the intestinal absorption predicted method. Meglumine antimoniate showed the lowest values for oral and intestinal absorption as was already known. However, all these compounds may theoretically act as P-glycoprotein substrates. Epoxy-*α*-lapachone showed a moderate distribution volume while epoxymethoxy-lawsone value was very low. On the other hand, the unbound fraction of epoxy-*α*-lapachone was lower than that of epoxymethoxy-lawsone (0.28 and 0.39, resp.). Meglumine antimoniate presented a very high unbound fraction (0.98), suggesting that this drug has the highest bioavailability. The ability of a drug to cross into the brain is an important parameter to consider in an attempt to reduce adverse effects or to improve the pharmacological activity of drugs that act within the brain. Two tests were conducted to predict this capability: blood-brain barrier (BBB) permeability and central nervous system (CNS) permeability. The latter is a more direct measure. The results indicated that both oxiranes might pass through into the brain.

Regarding the metabolism of these drugs, epoxy-*α*-lapachone is a substrate for CYP3A4. Surprisingly, epoxymethoxy-lawsone and meglumine antimoniate are not, at least theoretically, substrates for cytochrome P450 enzymes. Besides that, oxiranes may act as inhibitors of CYP1A2. Considering the toxicity, according to Table 1, epoxymethoxy-lawsone could have a mutagenic potential (AMES positive test) but it seems to be better tolerated than epoxy-*α*-lapachone (higher Maximum Tolerated Dose).

### 3.2. Histopathological Analysis of Healthy Mice Treated with Oxirane Derivatives and Meglumine Antimoniate

**Figure 2:**
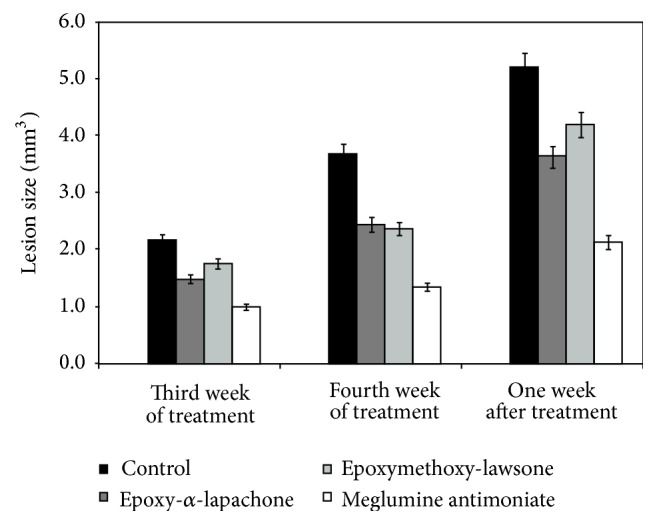
Treatment of infections in mice caused by* Leishmania* (*L.*)* amazonensis*. BALB/c mice were inoculated subcutaneously, in the left footpad, with 1.0 × 10^4^ promastigotes. After 4 weeks of infection, the mice were treated weekly with 22.7 mg/Kg/day (1.9 *μ*M/Kg/day) of epoxy-*α*-lapachone (dark grey square), 11.4 mg/Kg/day (1.2 *μ*M/Kg/day) of epoxymethoxy-lawsone (light grey square), and 22.7 mg/Kg/day (4.1 *μ*M of Sb^5+^/Kg/day) of meglumine antimoniate (white square), administered subcutaneously with five animals per group. Negative controls were treated with a mix of DMSO : propylene glycol : saline with higher concentration of propylene glycol (1 : 12 : 7) (black square). Lesion sizes (mm^3^) were measured weekly and the results of third and fourth weeks of treatment and one week after treatment are shown. The data are presented by the mean and standard deviation (SD), from five mice.

Histopathological analysis was performed in healthy BALB/c mice following a previous assay where we showed that these oxirane compounds and meglumine antimoniate were able to control the size of the lesion in mice infected with* L. (L.) amazonensis*. The largest difference was observed one week after the treatment whose main results are summarized in Figure 2. The most effective doses were selected to assess the tissue-specific toxicity. Epoxymethoxy-lawsone was as effective as epoxy-*α*-lapachone even when tested at a lower concentration. The best results were obtained when the reference drug was used.

In general, the drugs were able to induce changes in all tissues analyzed. The most affected organs were lung, heart, and brain. Epoxymethoxy-lawsone was the most toxic to the lung tissue, while most severe damage to the heart and brain was provoked by epoxy-*α*-lapachone. As expected, meglumine antimoniate induced severe changes in heart tissue and curiously caused changes to the lung tissue (Table 2).

#### 3.2.1. Histopathological Analysis of the Lung

**Figure 3:**
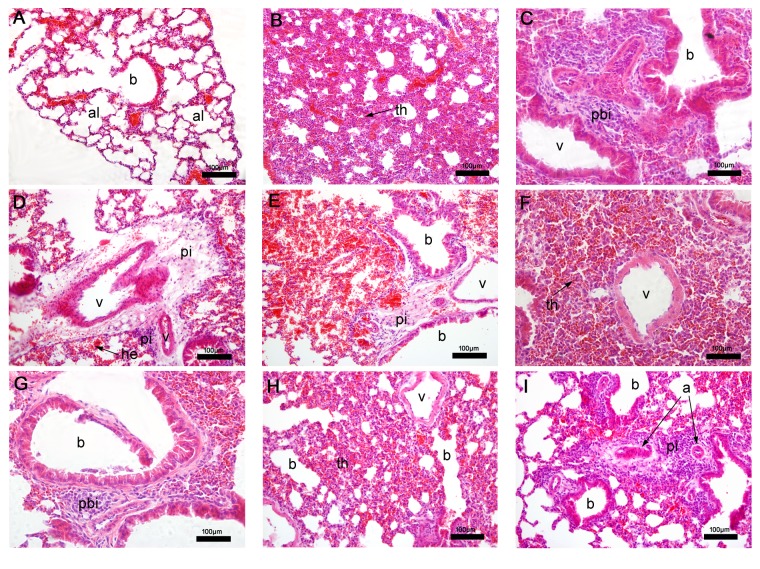
Histopathological analysis of mice lung tissue. Nontreated BALB/c mice (A) presenting normal aspect of alveoli and bronchiole and after treatment with epoxy-*α*-lapachone (B, C, D) and epoxymethoxy-lawsone (E, F, G) showing pulmonary alterations, including severe septal thickening, hemorrhage, presence of peribronchiole, and perivascular infiltration. After meglumine antimoniate treatment (H, I), moderate septal thickening and severe perivascular infiltration are observed. Lung tissues were stained with hematoxylin and eosin. Tissue images are representative of 10-fragment analysis by animals (*n* = 6), for each treatment group. Alveoli (al), bronchiole (b), hemorrhage (he), peribronchiole (pbi), perivascular infiltration (pi), pulmonary vein (v), and septal thickening (th).

As expected, in lung tissue of nontreated mice a regular structure of alveoli and alveolar septa was observed (Figure 3(A)). In BALB/c mice, treatment with epoxy-*α*-lapachone and epoxymethoxy-lawsone caused severe septum thickening and the appearance of mononuclear inflammatory infiltrates around the bronchiole and pulmonary veins, suggesting an interstitial pneumonia (Figures 3(B)–3(D)). Extensive areas of hemorrhage were found in the epoxymethoxy-lawsone treated group (Figures 3(E)–3(G)). The reference drug, meglumine antimoniate, induced similar changes such as moderate septum thickening (Figure 3(H)) and both severe peribronchiolar and perivascular infiltrates (Figure 3(I)).

#### 3.2.2. Histopathological Analysis of the Heart

**Figure 4:**
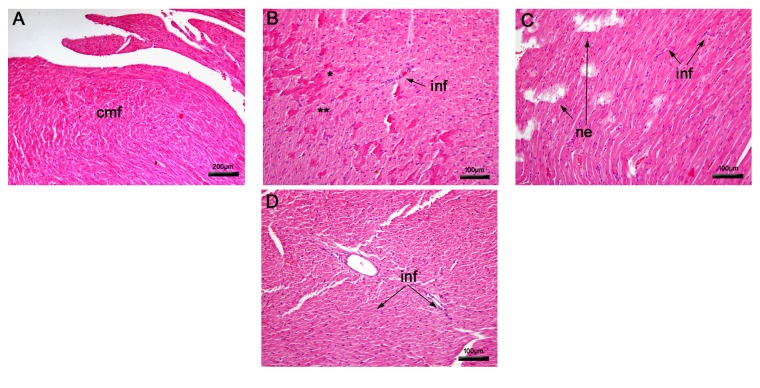
Histopathological analysis of mice heart tissue. Nontreated BALB/c mice presented normal structure (A) and after treatment with epoxy-*α*-lapachone (B) showed regenerated (*∗*) and degenerated cardiac fibers (*∗∗*) and epoxymethoxy-lawsone resulted in diffuse necrosis areas (C). A difference in the necrosis pattern was noted. In the case of epoxy-*α*-lapachone there is a more degenerative diffuse necrosis with focal areas of fiber regeneration while with epoxymethoxy-lawsone areas of severe necrosis are seen, but there are regions where the fiber structure is still preserved. Meglumine antimoniate caused moderate mononuclear infiltrates (D). Heart tissues were stained with hematoxylin and eosin. Tissue images are representative of 10-fragment analysis by animals (*n* = 6), for each treatment group. Cardiac muscular fibers (cmf), necrosis (ne), and infiltrates (inf).

A normal cardiac structure with branching fibers, central nuclei, and intercalated discs was observed in nontreated animal tissues (Figure 4(A)). On the other hand, oxiranes induced parenchyma alterations, such as severe-diffuse interstitial necrosis, leading to a degeneration of cardiac fibers (Figures 4(B) and 4(C)). In addition, a difference in the pattern of necrosis was observed. In the case of epoxy-*α*-lapachone there is a more degenerative diffuse necrosis with focal areas of fiber regeneration, while with epoxymethoxy-lawsone areas of severe necrosis were noted, but there are regions where the fibers structures are still preserved. Treatment with meglumine antimoniate also showed severe necrosis (not shown). Mononuclear infiltrates were noted in all treatments ranging from severe in meglumine antimoniate and epoxymethoxy-lawsone to severe-diffuse in epoxy-*α*-lapachone (Figures 4(B)–4(D)).

#### 3.2.3. Histopathological Analysis of the Kidney

**Figure 5:**
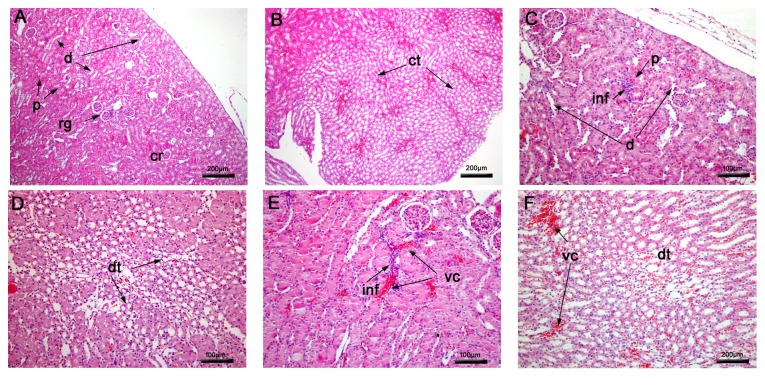
Histopathological analysis of mice kidney tissue. Nontreated BALB/c mice showed regular cortical and medullar regions (A, B) and after treatment with epoxy-*α*-lapachone showed mononuclear infiltrates near the proximal (p) tubules in focal areas of the cortical region and destructed epithelial lining of distal (d) convoluted tubules (C, D). Degenerate collector tubules were observed in diffuse areas of the medullar region. Epoxymethoxy-lawsone produced mononuclear infiltrates and vascular congestion in diffuse areas (E) and focal areas of vascular congestion and degenerate collector tubules (F). Kidney tissues were stained with hematoxylin and eosin. Tissue images are representative of 10-fragment analysis by animals (*n* = 6), for each treatment group. Collector tubules (tc), degenerate collector tubules (dt), epithelial lining of distal convoluted tubules (d), infiltrates (inf), molecular region (mr), proximal convoluted tubules (p), renal glomerulus (rg), and vascular congestion (vc).

As expected, in nontreated mice a normal structure with preserved distal and proximal convoluted tubules, intact renal glomerulus, and collector tubules was observed (Figures 5(A) and 5(B)), but both oxiranes caused several alterations in kidney tissue. Disorganization of the proximal and distal tubules was associated with all treatments; however, the most severe damage was provoked by epoxy-*α*-lapachone followed by meglumine antimoniate (moderate) and epoxymethoxy-lawsone (mild) (Figures 5(C) and 5(E)). We have detected severe destructed epithelial lining of distal convoluted tubules in the cortical area when mice were treated with oxiranes (Figures 5(C) and 5(E)). Mild-to-moderate cortical and medullar vascular congestion was found with the oxirane compounds (Figure 5(E)). Cortical and medullar infiltrates were related to all drugs ranging from moderate to severe (Figures 5(C)–5(F)). Additionally, epoxymethoxy-lawsone provoked severe vascular congestion in the medullar area (Figure 5(F)).

#### 3.2.4. Histopathological Analysis of the Liver

**Figure 6:**
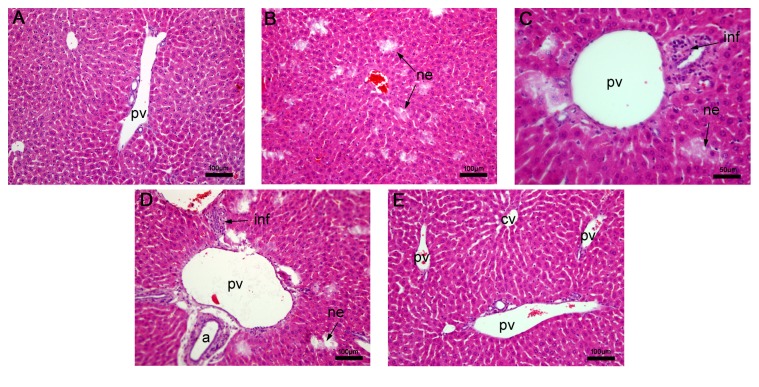
Histology analysis of the liver. (A) Nontreated BALB/c mice presenting normal aspect. Liver sections of mice treated with epoxy-*α*-lapachone showing diffuse areas of necrosis around the portal space and hepatic parenchyma and mononuclear infiltrates near portal vein and biliary ducts (B, C). In BALB/c mice treated with epoxymethoxy-lawsone triad portal space focal necrosis and infiltrates around biliary ducts and arterioles were noted (D, E). Mice treated with meglumine antimoniate retained the hepatic parenchyma with regular structure. Liver tissues were stained with hematoxylin and eosin. Tissue images are representative of 10-fragment analysis by animals (*n* = 6), for each treatment group. Arterioles (a), central vein (cv), infiltrates (inf), necrosis (ne), and portal vein (pv).

Liver tissue obtained from nontreated mice presented hepatocytes, sinusoidal capillaries, portal space, and central vein with regular structure (Figure 6(A)). Parenchymal damage was observed in both oxiranes treatments. The most significant changes detected with oxirane derivatives were necrosis and increased cellularity (Figures 6(B)–6(D)). These changes were more notable when epoxy-*α*-lapachone was used (Figures 6(B) and 6(C)). A mild hemorrhage and edema were also found in this group. No alterations were detected in the antimonial treated group (Figure 6(E)).

#### 3.2.5. Histopathological Analysis of the Brain/Cerebellum

**Figure 7:**
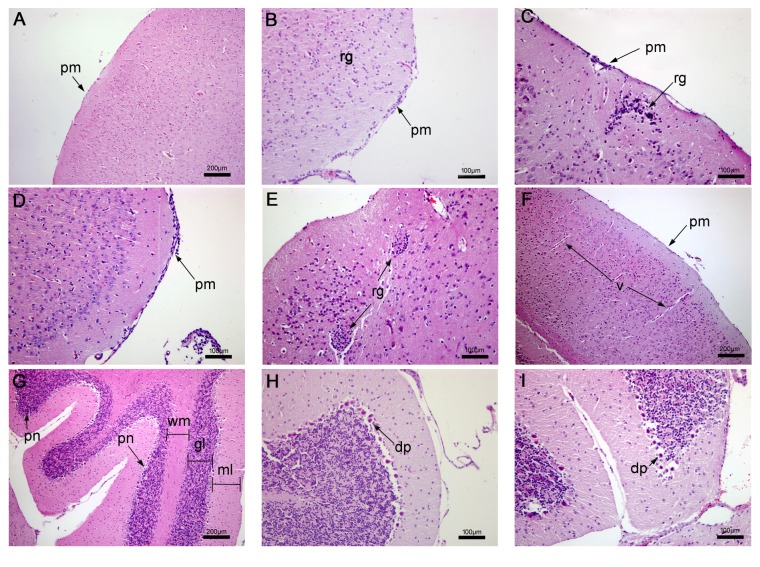
Histopathological analysis of mice brain and cerebellum. A regular architecture of the cerebral and cerebellar cortex of nontreated BALB/c mice is shown (A, G). Treated mice with epoxy-*α*-lapachone (B, C) and epoxymethoxy-lawsone (D, E) showed thickening of pia mater and reactive gliosis. No alterations were observed. Meglumine antimoniate in the cortex (F). Cerebellum of mice treated with oxiranes showed degeneration in Purkinje neurons (H, I). Alterations detected in brain and cerebellum of animals treated with oxiranes indicated a blood-brain barrier disruption. Brain tissues were stained with hematoxylin and eosin. Tissue images are representative of 10-fragment analysis by animals (*n* = 6), for each treatment group. Cortex region (cr), degenerate Purkinje neuron (dp), granular layer (gl), infiltrates (inf), molecular layer (mL), pia mater (pm), Purkinje neuron (pn), reactive gliose (rg), vein (v), and white matter (wm).

Brain and cerebellum of nontreated mice showed regular architecture of the cerebral and cerebellar cortex (Figures 7(A) and 7(G)). In BALB/c mice treated with epoxy-*α*-lapachone were observed thickening of the pia mater associated with intense diffuse infiltrates of the cortex and severe-diffuse reactive gliosis, suggesting an increase in the number of glial cells, mainly astrocytes (Figures 7(B) and 7(C)). The group treated with epoxymethoxy-lawsone showed similar but less severe alterations. Infiltrates in the cortex area were discrete in mice of this group (Figures 7(D) and 7(E)). Cerebellum of mice treated with oxiranes showed degeneration in Purkinje neurons (Figures 7(H) and 7(I)). No changes were observed in brain or cerebellum of mice treated with meglumine antimoniate (Figure 7(F)). The changes detected in brain and cerebellum of animals treated with oxiranes indicated a blood-brain barrier disruption, showing that these molecules are able to pass through into the central nervous system (SNC).

## 4. Discussion

New approaches in the chemotherapy of* Leishmania* spp. infections are well studied in murine models. However, drug efficacy studies may overestimate the beneficial effect of the interventions. The approach applied here consists in an effort to identify and describe toxicity profile of a new class of synthetic compounds, the oxiranes, combining an in silico prediction of pharmacokinetic parameters with histopathological analysis. Data obtained here with an inbred strain of mice, non-*Leishmania*-infected, emphasize the importance of monitoring and improving the understanding of the toxic effects on human tissues due to resemblance in physiology.

The present study shows strong evidence that the treatment with oxiranes epoxy-*α*-lapachone and epoxymethoxy-lawsone at dose of 22.7 mg/Kg/day and 11.4 mg/Kg/day, respectively, administrated daily from Monday to Friday, until 20 doses in healthy mice caused substantial histological changes in lung, heart, kidney, liver, and brain of healthy mice. The same protocol which had been previously applied to treat mice infected by* L. (L.) amazonensis* controlled the size of the lesion. In this context, analyses were based on the higher dose considered as the most effective in controlling progression of the lesion in mice. Further studies are necessary for the follow-up of animals in the weeks after treatment, to provide data about persistence or reversibility of the changes found.

The toxic effects of oxiranes detected in mice tissues may be explained by the chemical nature of these compounds and their putative pharmacokinetic effects as assessed. Oxiranes are synthetic compounds derived from natural naphthoquinones and bioactive against* Trypanosoma cruzi* [26] and* Leishmania* spp. [24]. Epoxy-*α*-lapachone and epoxymethoxy-lawsone were based on *α*-lapachone and 2-hydroxy-1,4-naphthoquinone (lawsone), respectively [27]. For this reason, such molecules might share the toxic properties already described for these quinones.

The injuries observed may be due to their high distribution in the tissues and the oxidation/reduction mechanism earlier described for quinones. Quinones are oxidants agents and electrophiles, and the relative contribution of these properties to both toxic and therapeutic activities can mainly be influenced by the effects of their substituents and the characteristics of the quinone core [17]. The redox cycling of quinones may be initiated by either a one- or two-electron reduction. The one electron reduction of quinones is catalyzed by NADPH-cytochrome P450 reductase and other flavoprotein enzymes, leading to unstable semiquinones. Semiquinones transfer electrons to molecular oxygen (O_2_) and return to their original quinoidal structure, thus generating a superoxide anion radical (  O_2_^−•^). Superoxide can be converted into hydrogen peroxide (H_2_O_2_) via a superoxide dismutase- (SOD-) catalyzed reaction, followed by the formation of a hydroxyl radical (HO^•^) through the iron catalyzed reduction of peroxide via the Fenton reaction (Supplemental file, in Supplementary Material available online at https://doi.org/10.1155/2017/9840210). All of these reactive oxygen species are powerful oxidizing agents and are probably responsible for damage to macromolecules such as DNA, proteins, and lipids, leading to oxidative stress and apoptosis in the cells [13, 17, 18, 28]. Thus, it is possible that some of these pathways would be overactivated in the lung due to a high concentration of molecular oxygen and iron ion present in pulmonary alveoli, which could explain the extensive damage found in this organ.

Another mechanism for signs of toxicity found in tissues exposed to naphthoquinones is that these compounds act as electrophiles forming covalent bonds with nucleophilic functions in biological molecules in an arylation reaction. When the nucleophile is a thiol group, the reaction generates a thioether, which is usually stable [28]. Therefore, the molecular basis for the quinone cytotoxicity has been attributed to the alkylation of essential protein thiol and amine groups as well as the oxidation of essential protein thiols by activated reactive oxygen species [17].

Our results detected vascular changes in lung as severe-diffuse hemorrhage and intense perivascular infiltrates, besides vascular congestion in both the cortical and medullar areas and a mild medullar hemorrhage in kidney of mice treated with epoxymethoxy-lawsone.

The fact that meglumine antimoniate has caused important changes in mice tissues, with relevant toxic effects in both heart and lung tissues, is a matter of concern. Pentavalent antimonials have been used for decades, and complaints related to lung function by the patients are very rare. In addition, the most toxic effects refer to heart function, particularly ventricular repolarization disorders and an increase of corrected QT interval (QTc) [5]. It was demonstrated that both trivalent potassium antimony (III) tartrate and sodium stibogluconate, regardless of their different oxidation states, increased cardiac calcium currents at therapeutic concentrations, whereas three major cardiac potassium currents (*I*_Kr_, *I*_Ks_, and *I*_K1_) were not affected. Cardiac calcium currents were especially sensitive to the trivalent antimony compound. According to authors, as calcium currents regulate the plateau phase of the cardiac action potential and an increased amplitude provokes a delay in cardiac repolarization, this finding may explain the occurrence of development QTc prolongation which can lead to ventricular tachycardia,* torsades de pointes,* and other arrhythmias in patients treated with antimonial drugs [29]. Indeed, we have evidence that meglumine antimoniate induced severe necrosis in murine cardiac tissue with presence of very intense mononuclear infiltration. These data suggest that such foci of necrosis may be responsible, in part, for the remarkable electrocardiographic changes during treatment with antimonials; however, supplementary assays are needed to prove this hypothesis.

Data obtained by in silico analysis indicate a possible application of oxirane compounds by oral or topical route. This might allow exploration of alternative ways for the administration of these compounds aimed at reducing their toxic effects. Furthermore, a high distribution predicted for oxiranes in brain tissues suggested by this analysis was confirmed by the histological changes found in the brain and cerebellum of mice, indicating that these compounds pass into the SNC. Findings such as inflammation and thickening of the pia mater and reactive gliosis indicate encephalitis, which was most severe and intense with epoxy-*α*-lapachone treatment. This relevant information must be considered in any formulation as a guide to reduce side effects and toxicity.

On the other hand, the negative prediction of toxic effects on hepatocytes was not confirmed. We have detected several foci of severe-to-moderate necrosis and intense inflammatory infiltrate in hepatic tissue of animals treated with oxiranes. It is demonstrated that the application of computational technologies to predict kinetic properties and to identify the toxicity potential based on the physicochemical and structural characteristics of the chemical substances raises relevant theoretical issues but must be further assessed by biological assays.

## 5. Conclusions

In conclusion, data presented here add evidence for toxicity induced by oxiranes and meglumine antimoniate, characterized by histological changes in different vital organs of healthy mice. Toxic and adverse effects of drugs are related to several factors such as dose, time, and frequency of exposure, route of administration, and pharmacokinetic parameters. Nonetheless, an active molecule that initially has been shown to be toxic should not be discarded before considering alternatives such as reduction of dose or duration of treatment, combined use with other drugs, and study of different formulations. The best example is amphotericin B, a very active molecule against different microorganisms but also very toxic to mammalian cells in which concepts of drug delivery systems involving liposomal formulations with lower toxicity have been applied [30].

## Supplementary Material

Proposed mechanisms for the toxicity of oxiranes derived from natural naphthoquinones.

## Figures and Tables

**Table 1:** In silico pharmacokinetic and toxicological parameters for oxirane compounds and meglumine antimoniate.

Predicted properties	Epoxy-*α*-lapachone	Epoxy-methoxy-lapachol	Meglumine antimoniate	Interpretation
*Absorption*				
Caco2 permeability	1.595	1.584	−0.443	>0,90
Intestinal absorption	100	100	27.93	% absorbed
Skin Permeability	−3.242	−3.249	−2.893	>−2.5 low skin permeability
P-Glycoprotein substrate	Yes	Yes	Yes	Yes/no
P-Glycoprotein inhibitor	No	No	No	Yes/no
*Distribution*				
Volume of distribution	0.201	−0.012	−0.35	Low: <−0.15; high: >0.45
Fraction unbound (human)	0.283	0.392	0.985	0.0 to 1.0
BBB permeability	0.325	0.260	−1.287	Low: <−1/high: >0.30
CNS permeability	−2.087	−2.014	−4.761	Positive: >−2; negative: <−3
*Metabolism*				
CYP3A4 substrate	Yes	No	No	Yes/no
CYP2D6 substrate	No	No	No	Yes/no
CYP3A4 inhibitor	No	No	No	Yes/no
CYP2D6 inhibitor	No	No	No	Yes/no
CYP1A2 inhibitor	Yes	Yes	No	Yes/no
*Excretion*				
Total clearance	0.07	0.197	−0.154	log mL/min/kg
*Toxicity*				
AMES toxicity	No	Yes	No	Yes/no
Max. tolerated dose (human)	0.536	0.925	0.947	log mg/kg/day
Oral Rat Acute Toxicity (LD50)	1.975	1.962	1.184	mol/kg
Oral Rat Chronic Toxicity (LOAEL)	1.728	2.290	1.218	log mg/kg_bw/day
Hepatotoxicity	No	No	No	Yes/no
Skin sensitisation	No	No	No	Yes/no

**Table 2:** Semiquantitative analysis of histological changes in tissues of mice treated with oxiranes derivate of natural 1,4-naphthoquinones and meglumine antimoniate.

Changes	Epoxy-*α*-lapachone	Epoxymethoxy-lawsone	Meglumine antimoniate
*Lung*			
Thickening of the alveolar septa	+++	+++	++
Peribronchiolar infiltrates	+++	+++	+++
Perivascular infiltrates	++	++++	+++
Hemorrhage	++	++++	+
*Heart*			
Necrosis	++++	+++	+++
Degeneration of cardiac fibers	++++	+++	0
Mononuclear infiltrates	++++	+++	+++
*Kidney*			
Disorganization of the proximal and distal tubules	+++	++	++
Destructed epithelial lining of distal convoluted tubules	+++	+++	0
Disorganization collecting tubules regions	+++	++	0
Cortical infiltrates	++	+++	++
Medullar infiltrates	+++	++	++
Cortical hemorrhage	++	0	0
Medullar hemorrhage	+	+	0
Vascular congestion in the cortical area	+	++	0
Vascular congestion in the medullar area	+	+++	0
*Liver*			
Necrosis	+++	++	0
Mononuclear infiltrates	+++	+++	0
Hemorrhage	+	0	0
Edema	+	0	0
*Brain/Cerebellum*			
Mononuclear infiltrates in Pia Mater	++++	++	0
Disorganization of the cortical region	++++	++	0
Reactive gliosis	++++	++	0
Mononuclear infiltrates in parenchyma	++++	++	0
Degeneration in Purkinje Neuron	++++	+++	0

Grades were assigned using a subjective scale of 0 to ++++ for the different changes. 0 = absent, + = mild, ++ = moderate, +++ = severe, and ++++ = severe-diffuse.
