# Supplementary material for: Evidence for Tissue Toxicity in BALB/c Exposed to a Long-Term Treatment with Oxiranes Compared to Meglumine Antimoniate
Source: Biomed Res Int. 2017 Jul 17;2017:9840210. doi: 10.1155/2017/9840210 (PMC5535747; doi:10.1155/2017/9840210)
Supplement: Supplementary file 1 — Proposed mechanisms for the toxicity of oxiranes derived from natural naphthoquinones. [file 9840210.f1.docx]

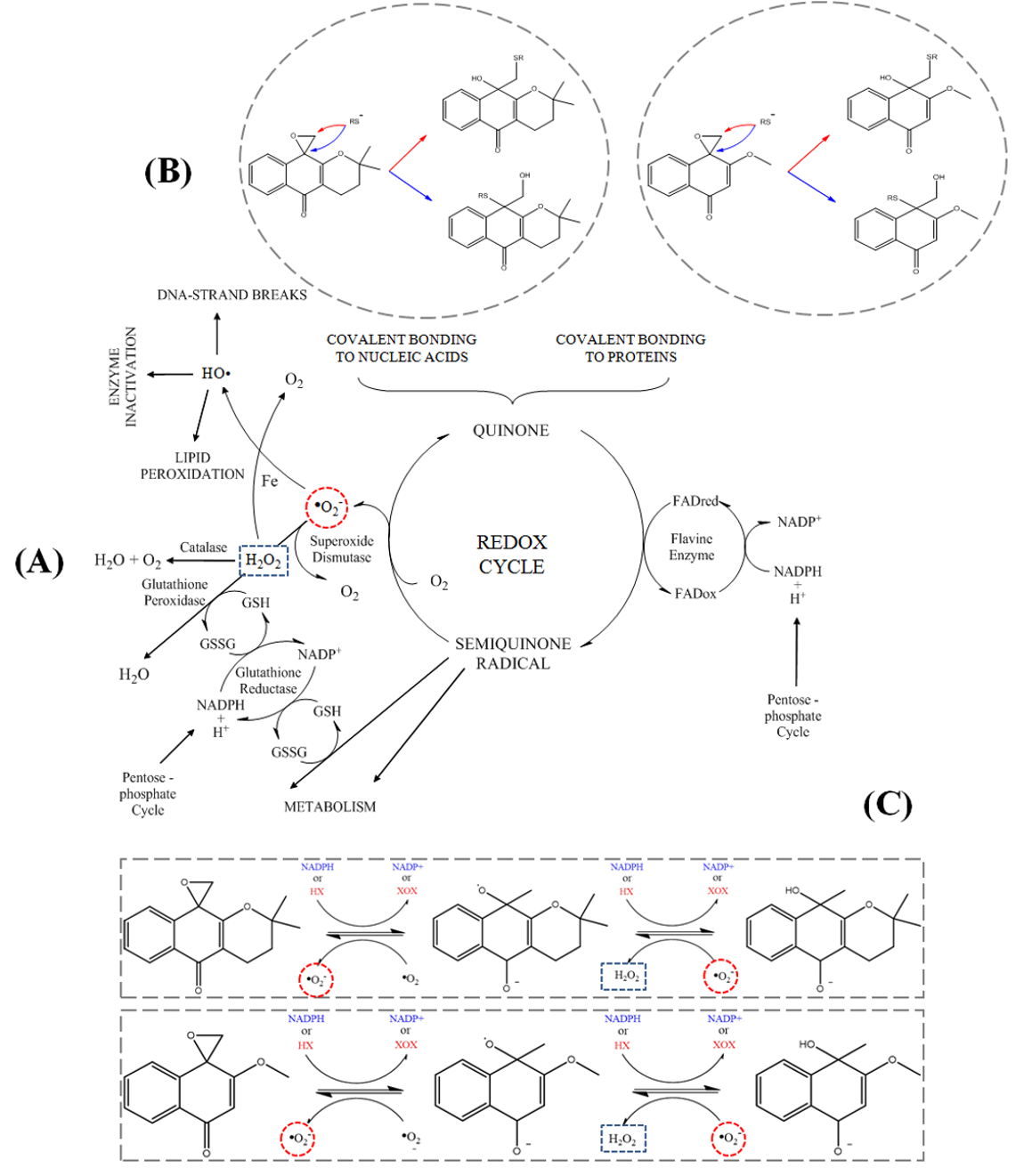


**Figure -** **(A)**: Overview of redox cycling of quinonoide compounds showing oxy radical formation and inactivation (adapted from Kappus 1986). The one electron reduction of quinones is catalyzed by NADPH-cytochrome P450 reductase and other flavoprotein enzymes, leading to unstable semiquinones which transfers electrons to molecular oxygen (O_2_), returning to their original quinoidal structure. A superoxide anion radical (•O_2_-) is generated and can be converted to hydrogen peroxide (H_2_O_2_) via a superoxide dismutase (SOD)-catalyzed reaction, followed by the formation of a hydroxyl radical (HO•) through the iron catalyzed reduction of peroxide via the Fenton reaction. **(B)**: Toxicity mechanism proposed (adapted from Kumagai 2012) is that these compounds may act as electrophiles forming covalent bonds with nucleophilic functions in biological molecules in an arylation reaction. When the nucleophile is a thiol group (represented by RS^-^), the reaction generates a thioether, which is usually stable. Differently from quinones, the oxiranes not suffer the called Michael addition due to the most electronegative part of these compounds is in the oxirane moiety. The thiol of cysteine (Cys) is the major redox-active and nucleophilic functional group in biological systems. This amino acid is a component of the redox-active peptide gluttathione (GSH) and many proteins. **(C)**: Another toxicity mechanism proposed (adapted from Kumagai 2012) to explain histological changes found in healthy animal tissues treated with oxiranes. Oxiranes are synthetic derived from natural naphthoquinone and may share the toxic properties already described for these quinones and might participate in the initiation of and the propagation of free radical chain reactions. Free radicals are reactive chemical species with an unshared electron that can be transferred to other species. When oxygen is involved in these reactions, it is reduced to reactive oxygen species (ROS), a common term used to describe superoxide anion radical (•O_2_-), hydroxyl radical (HO•), and hydrogen peroxide (H_2_O_2_). Epoxy-α-lapachone and epoxymethoxy-lawsone were used at dose of 22.7 mg/Kg/day and 11.4 mg/Kg/day, respectively, administrated daily from Monday to Friday, until 20 doses. The dotted shapes show superoxide anion radical (•O_2_-) and hydrogen peroxide (H_2_O_2_) generated in both A and C. HX/XOX: Hypoxanthine/Xanthine oxidase.
